# Supplementary material for: Functional connectivity development along the sensorimotor-association axis enhances the cortical hierarchy
Source: Nat Commun. 2024 Apr 25;15:3511. doi: 10.1038/s41467-024-47748-w (PMC11045762; doi:10.1038/s41467-024-47748-w)
Supplement: Supplementary file 1 — Supplementary Information [file 41467_2024_47748_MOESM1_ESM.pdf]

SUPPLEMENTARY FIGURES

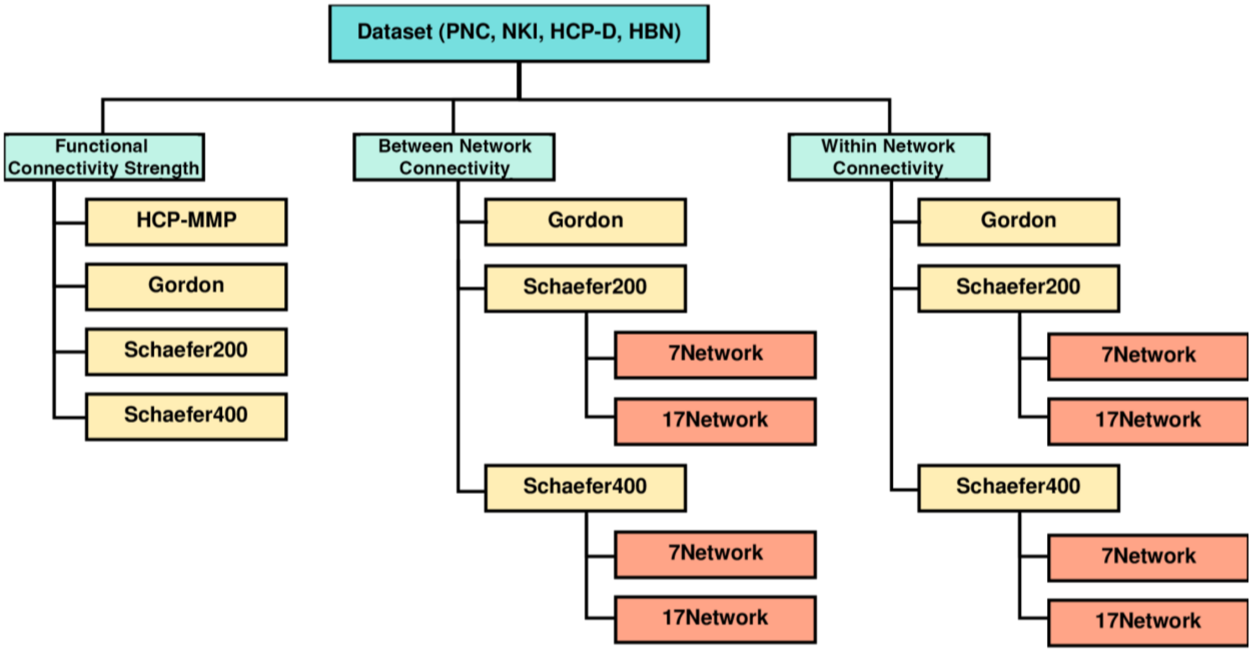

Supplementary Figure 1. Diagram depicting the parameter space for each dataset. Functional connectivity metrics were computed for each of the four datasets in this study, including Philadelphia Neurodevelopmental Cohort (PNC), Nathan-Kline Institute-Rockland Sample (NKI), Human Connectome Project: Development (HCP-D), and Healthy Brain Network (HBN). The primary functional connectivity metric was functional connectivity strength, computed as the mean edge strength of a given region and all other network regions. Secondary measures of functional connectivity were regional measures of average between- and within-network connectivity. Average between-network connectivity was calculated as the mean edge strength of a given region and all other network regions that are not in that region's community. Average within-network connectivity was calculated as the mean edge strength of a given region and all other network regions within that region's network community. The primary parcellation for all functional connectivity metrics was the Schaefer 200 atlas; the 7-network partition was the primary partition for between- and within-network analyses.[13,40] For functional connectivity strength, sensitivity analyses were conducted in three additional atlases: HCP-MMP, Gordon, and Schaefer 400 atlases.[40,91,92] Sensitivity analyses for between- and within-network connectivity were conducted in Gordon, Schaefer 200 (17-network solution), and Schaefer 400 (7- and 17-network solutions).

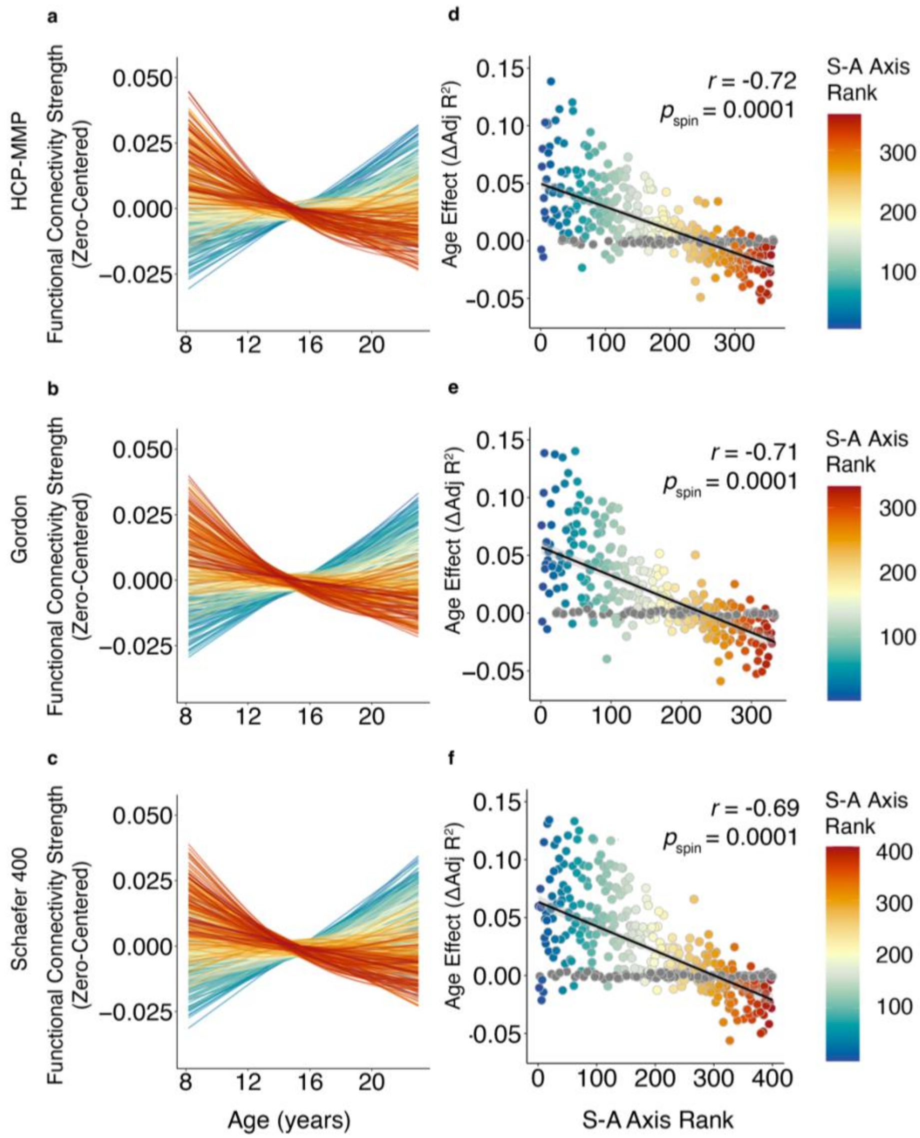

Supplementary Figure 2. Sensitivity analysis with secondary parcellations to examine functional connectivity strength development provides convergent results. We computed functional connectivity strength from concatenated resting-state and task fMRI for additional parcellations: HCP-MMP, Gordon, and Schaefer 400 atlases in all datasets. Sensitivity analyses with additional parcellations were completed in all datasets; representative results from sensitivity analyses in the PNC are depicted here. (**a-c**) Similar to our main findings using the primary parcellation (Schaefer 200 atlas), dissociable patterns of functional connectivity strength developmental trajectories can be seen along the S-A axis across all secondary parcellations. (**d-f**) The strong alignment between the age effect of functional connectivity strength and a given region's rank on the S-A axis is consistent across parcellations (HCP-MMP: r = -0.72, p_{spin} = 0.0001; Gordon: r = -0.71, p_{spin} = 0.0001; Schaefer 400: r = -0.69, p_{spin} = 0.0001). Spearman's rank correlations were used to quantify the association between S-A axis ranks and observed developmental effects with statistical significance determined using spin-based spatial permutation tests.

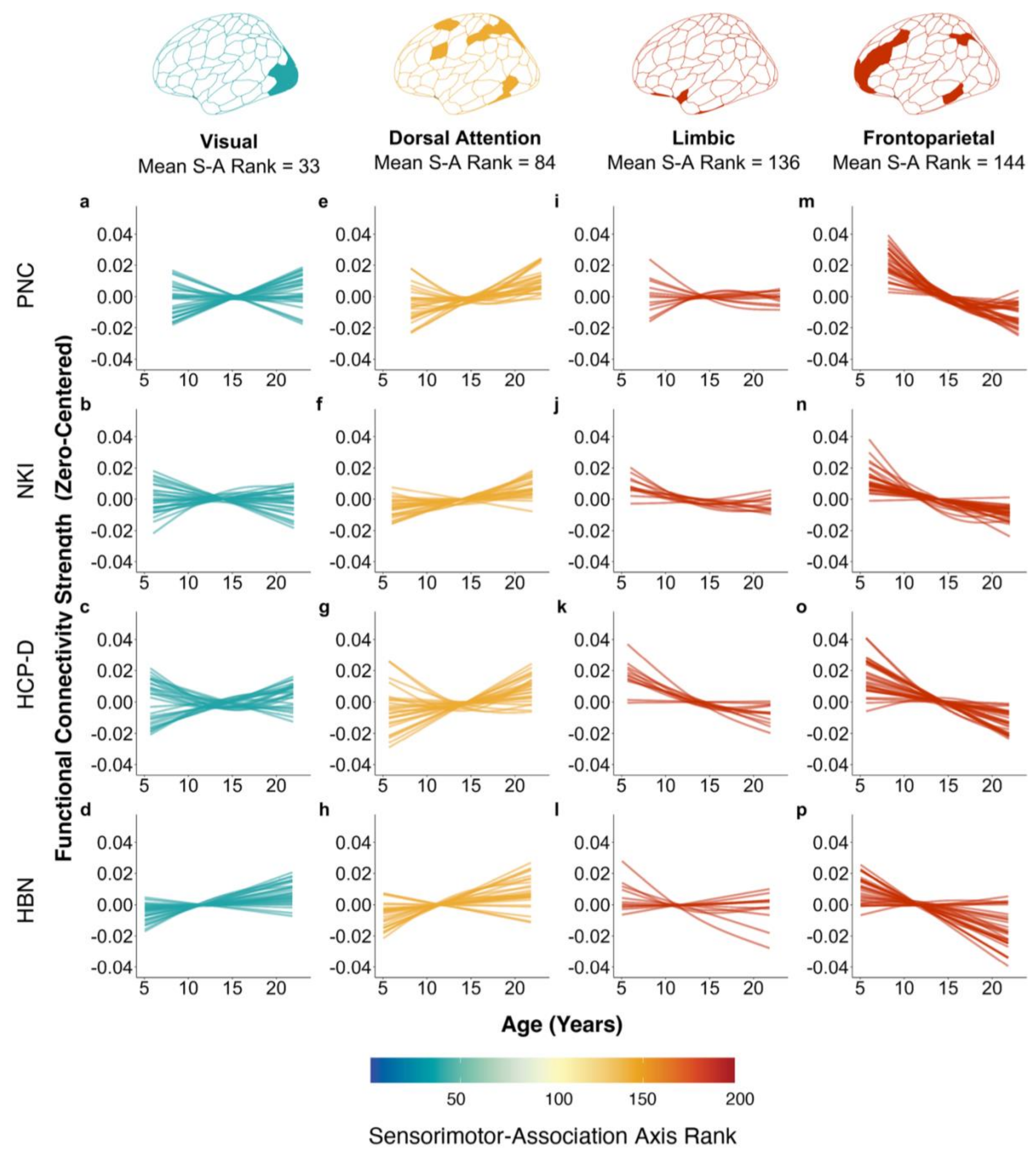

Supplementary Figure 3. Patterns of maturation across the sensorimotor-association axis for additional functional networks. (**a-p**) The plots display the developmental trajectories for regions from additional functional networks: (**a-d**) the visual, (**e-h**) dorsal attention, (**i-l**) limbic, and (**m-p**) fronto-parietal network. Each line represents an individual region's FC strength developmental trajectory (zero-centered), modeled using generalized additive models. Colors indicate the rank of a given region along the S-A axis.

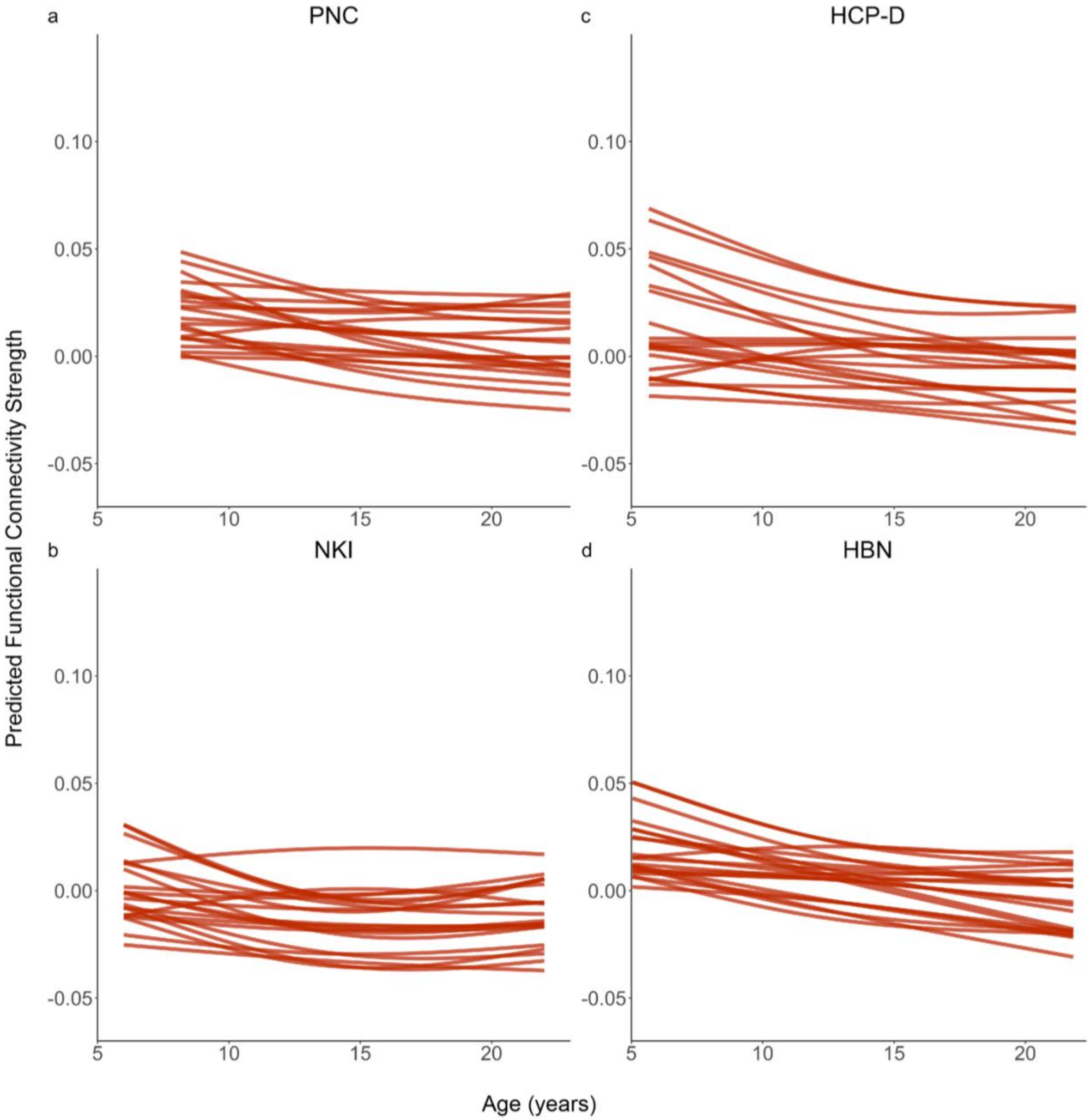

Supplementary Figure 4. Functional connectivity strength decreases in association regions with age. Developmental trajectories for predicted functional connectivity strength in the highest-ranking regions (rank > 180; most are part of default mode network) are shown for (a) PNC, (b) NKI, (c) HCP-D, and (d) HBN. Regions in the association pole show decreases in functional connectivity strength through development. A majority of regions display positive functional connectivity strength in childhood that becomes negative through adolescence. Several regions display increasingly negative functional connectivity strength with age; most of these regions were part of the default mode network. Each line represents the functional connectivity strength for each region throughout development, modeled using generalized additive models.

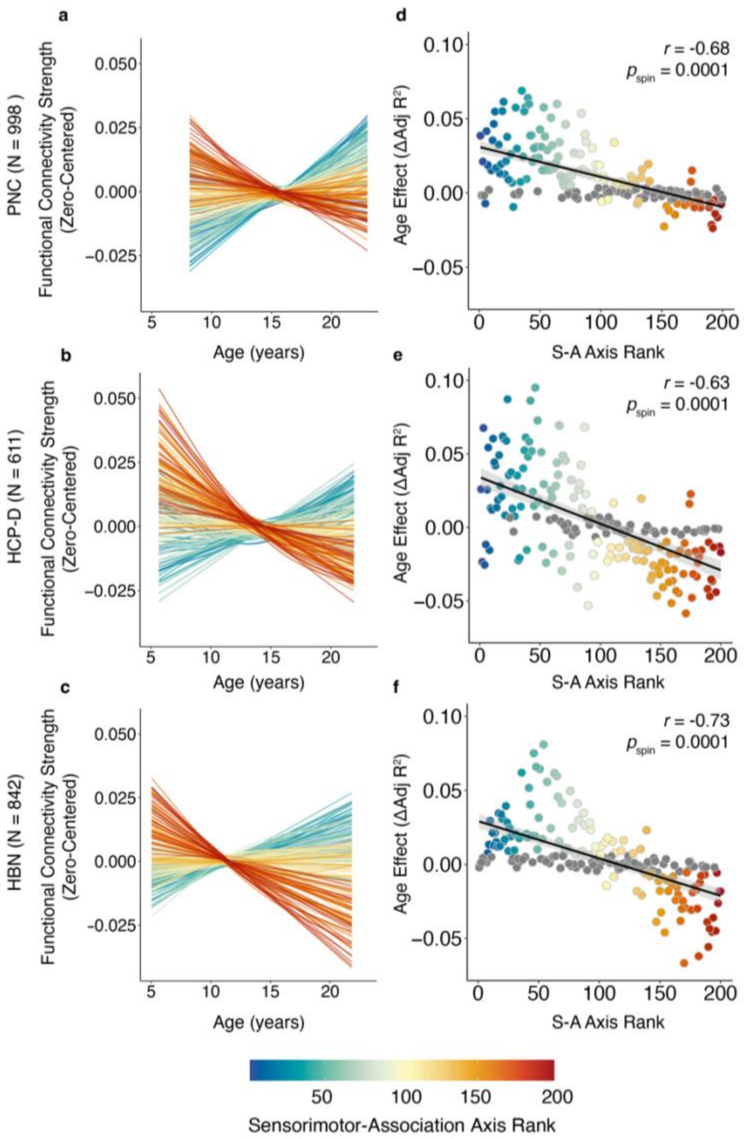

Supplementary Figure 5. Sensitivity analysis using only resting-state fMRI to examine developmental changes in functional connectivity strength provide convergent results. Consistent with our main findings using concatenated rest and task fMRI data, the development of rest-defined functional connectivity strength varies continuously along the S-A axis. (**a-c**) In the PNC, HCP-D, and HBN, regions in the sensorimotor pole generally show increases in functional connectivity strength whereas regions in the association pole show decreases in functional connectivity strength through development. Each line represents the functional connectivity strength (zero-centered) for each region throughout development, modeled using generalized additive models. (**d**) The rank of each region in the S-A axis explains the majority of variance in age effects in the PNC ($r = -0.68$, $p_{spin} = 0.0001$). These findings were replicated in additional independent datasets, including (**e**) HCP-D ($r = -0.63$, $p_{spin} = 0.0001$), and (**f**) HBN ($r = -0.73$, $p_{spin} = 0.0001$). The age effect of functional connectivity strength for each region (Schaefer 200) is plotted against the given region's rank in the S-A axis. Spearman's rank correlations were used to quantify the association between S-A axis ranks and observed developmental effects with statistical significance determined using spin-based spatial permutation tests. Note that axes were adjusted to best visualize all datasets; a total of 4 data points across all datasets were excluded for visualization.

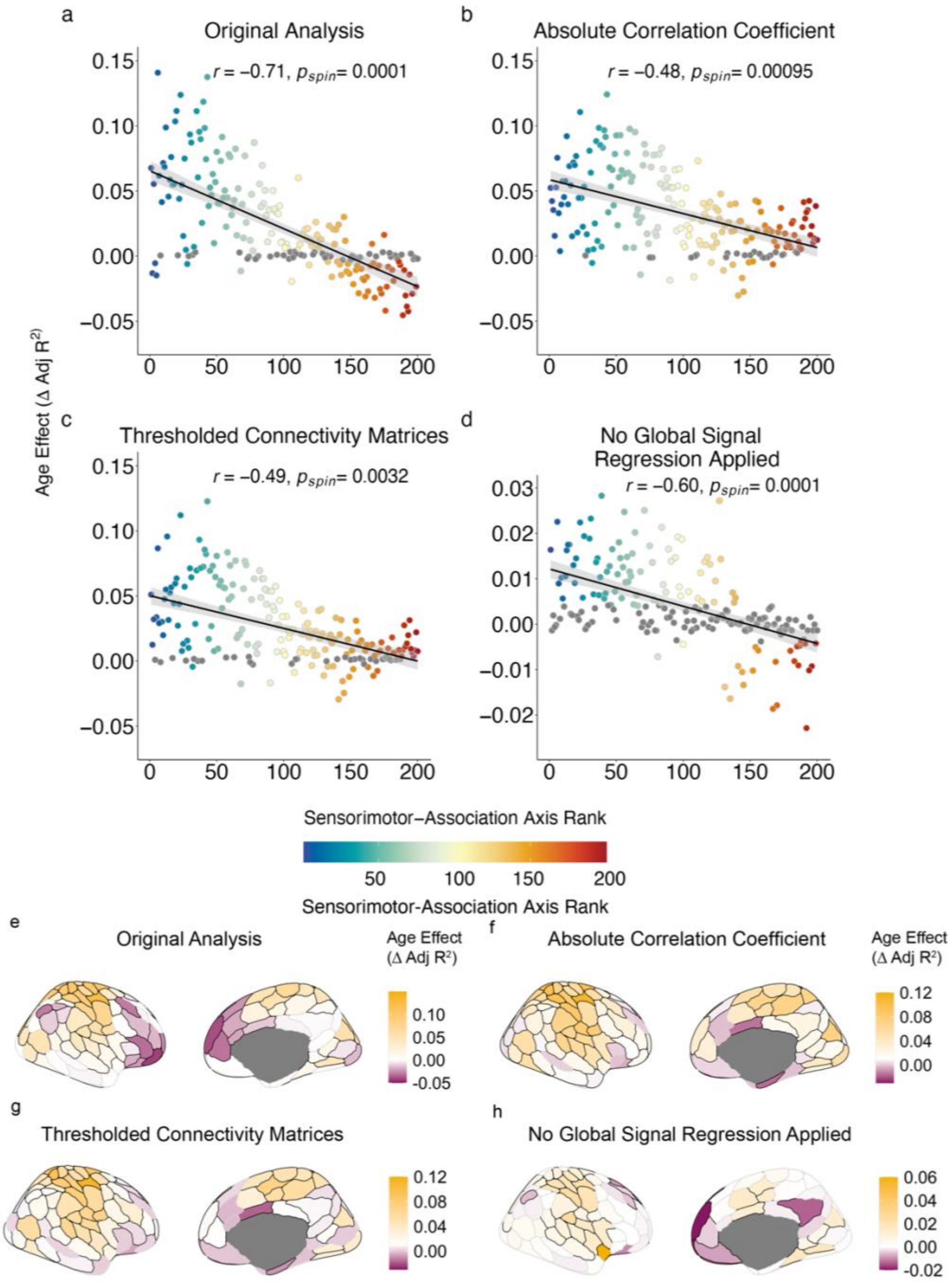

Supplementary Figure 6. Sensitivity analyses using absolute correlation, thresholded connectivity matrices, and no global signal regression to compute functional connectivity strength provide convergent results. (a) Original analysis from the PNC correlating the age effect of functional connectivity (FC) strength with sensorimotor-association axis rank is displayed for comparison to results of additional analyses. Sensitivity analyses in the PNC were conducted using FC strength computed (b) using the absolute value of the correlation coefficient as the measure of functional connectivity, (c) from thresholded connectivity matrices (including positive correlations only), and (d) without global signal regression. Consistent with our main findings using global signal regression and Pearson correlation, the development of FC strength varies continuously along the S-A axis. (b-d) The rank of each region in the

S-A axis is significantly associated with FC strength age effects in the PNC using absolute correlation (*r* = -0.48, p_{spin} = 0.00095), thresholded matrices (*r* = -0.49, p_{spin} = 0.0032), and no global signal regression (*r* = -0.60, p_{spin} = 0.0001). In association regions at the top of the S-A axis (primarily in the default network) in (b) and (c), negative age effects are abolished and are found to be positive. The age effect of FC strength remains negatively associated with S-A axis rank even after excluding GSR (d), suggesting that GSR played a minimal role in our findings. Maps of mean functional connectivity strength across all ages are displayed for (e) the original analysis in PNC and analyses (f) using the absolute value of the correlation coefficient as the measure of functional connectivity, (g) using thresholded connectivity matrices, and (h) excluding global signal regression. Spearman's rank correlations were used to quantify the association between S-A axis ranks and observed developmental effects with statistical significance determined using spin-based spatial permutation tests.

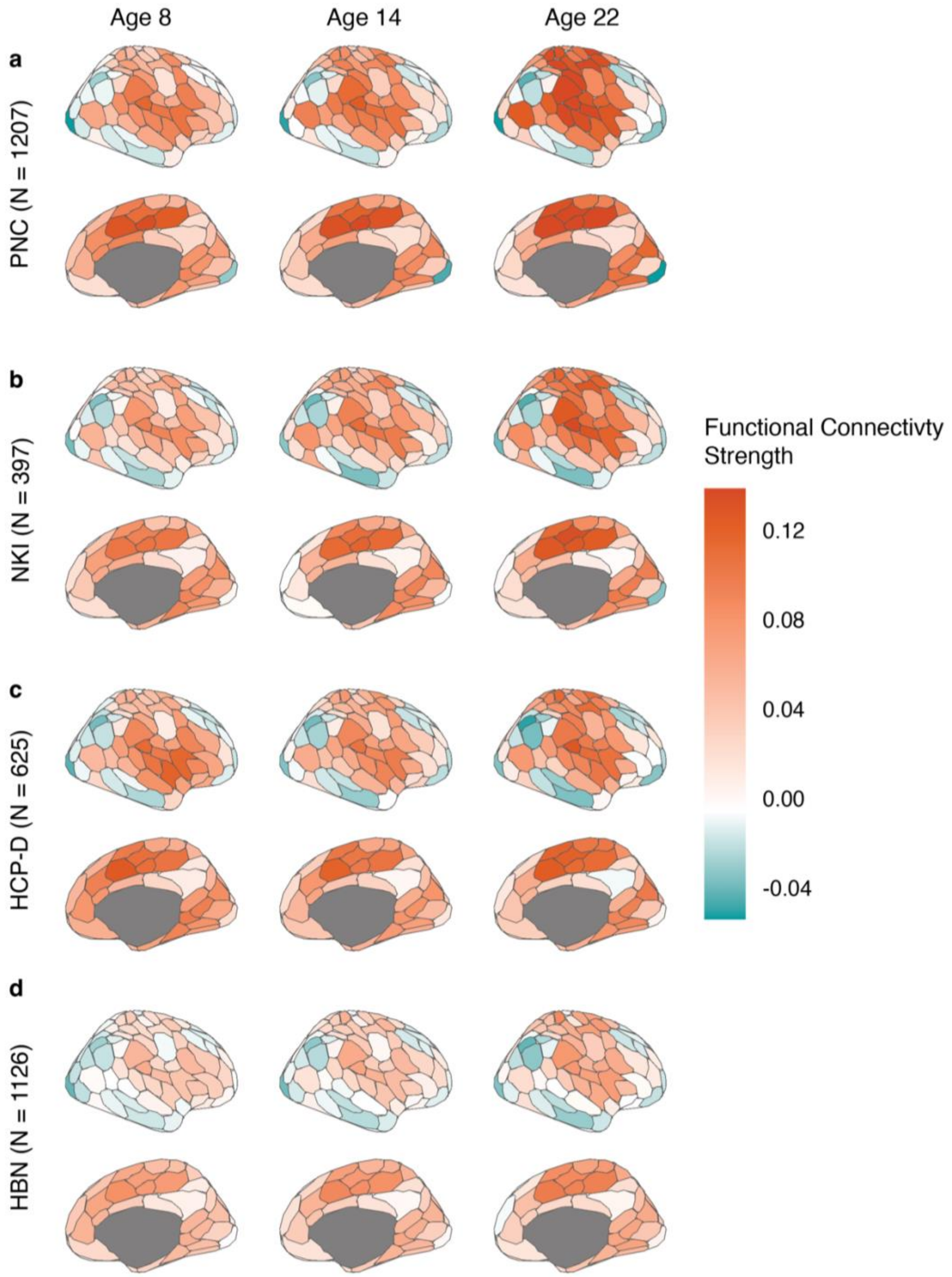

Supplementary Figure 7. The spatial distribution of functional connectivity strength is highly similar across all four datasets and is refined with age. The refinement of functional connectivity (FC) strength across the cortex appears highly similar across all four datasets. In the Philadelphia Neurodevelopmental Cohort (PNC), (a) the fitted values of FC strength predicted from regional generalized additive models are highest in somatomotor cortices in childhood and increase in these cortical areas with age. In contrast, fitted FC strength in association cortices is lower in childhood and tends to decrease with age. This decrease results in negative FC strength values in transmodal association cortices by early adulthood, suggestive of weakly anti-correlated connectivity with most brain regions. Similar spatial patterns of FC strength are seen in (**b**) Nathan Kline Institute-Rockland (NKI), (**c**) Human

Connectome Project: Development (HCP-D), and (**d**) Healthy Brain Network (HBN). In **a-d,** the predicted value of FC strength in each region is shown at ages 8, 14, and 22 across the four datasets. Generalized additive models were fitted independently for each cortical region and used to predict the fitted value of FC strength at each age. Each row of plots corresponds to results from a given dataset. Columns represent the FC strength map at each age. Results are shown in the Schaefer 200 atlas.

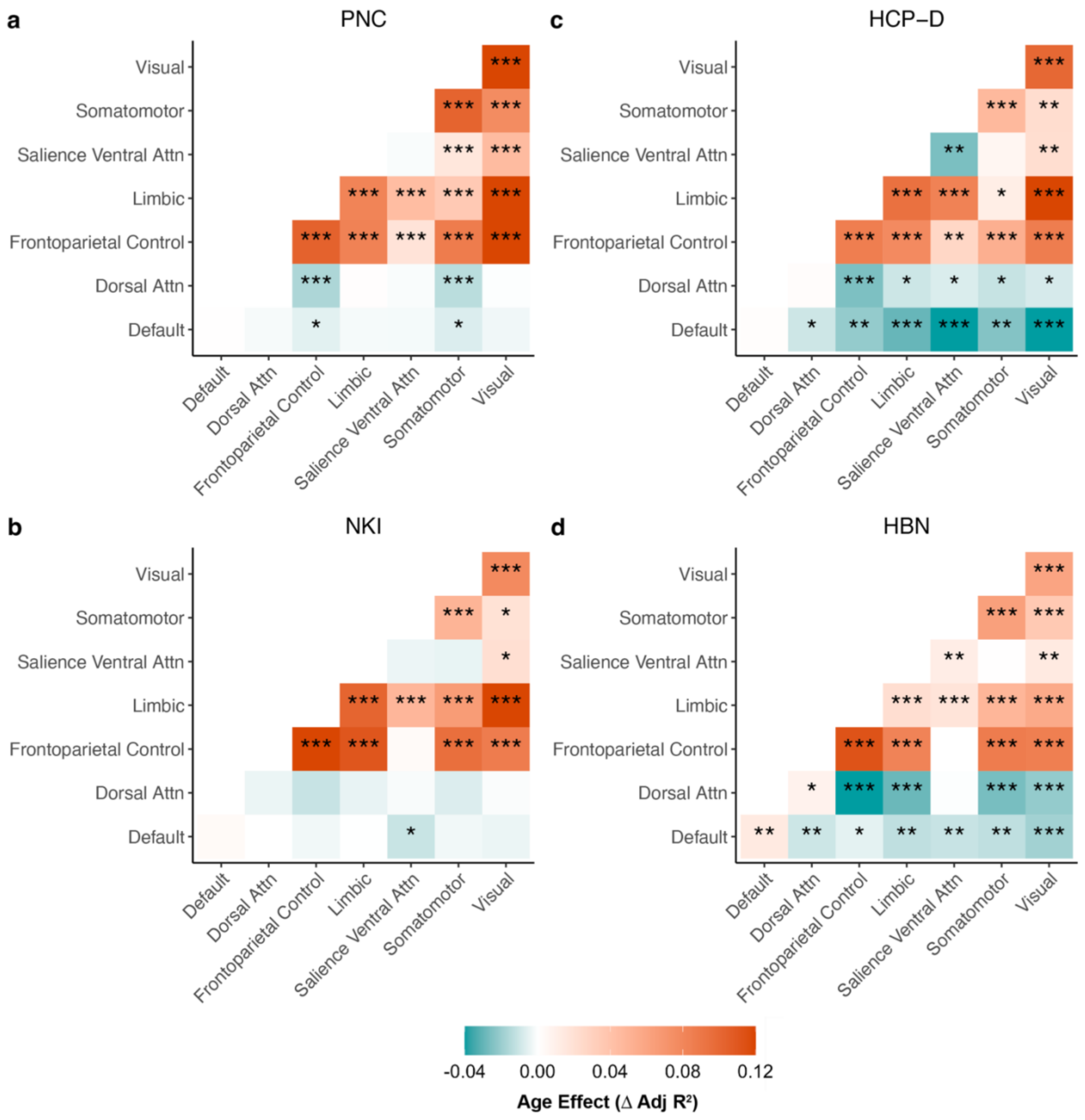

Supplementary Figure 8. Developmental changes in connectivity between specific pairs of networks vary in direction and magnitude. The age effect of average connectivity between each pair of networks, derived from the Yeo 7 network solution, is displayed in each colored box. Within-network connectivity in visual, somatomotor, limbic, and fronto-parietal networks consistently increase with age. Connectivity within the default mode network also increases in HBN. Sensorimotor networks with the lowest average

S-A ranks generally strengthen in between-network connectivity. The visual network shows most prominent increases with somatomotor, limbic, and fronto-parietal networks. The somatomotor network displays similar increases with age. In the higher order networks, limbic and fronto-parietal networks strengthen in between-network connectivity with all other networks except for default and dorsal attention networks. The highest ranked network – the default mode – tends to segregate from all other networks with age. Colored boxes represent the age effect of connectivity between each pair of networks, as computed by change in adjusted R^2 between a full model and reduced generalized additive model with no age term. FDR-corrected q-values: *** $q < 0.0001$, ** $q < 0.001$, * $q < 0.05$. Pairs of networks that do not display significant change in connectivity over development are colored in white ($Q_{FDR} > 0.05$).

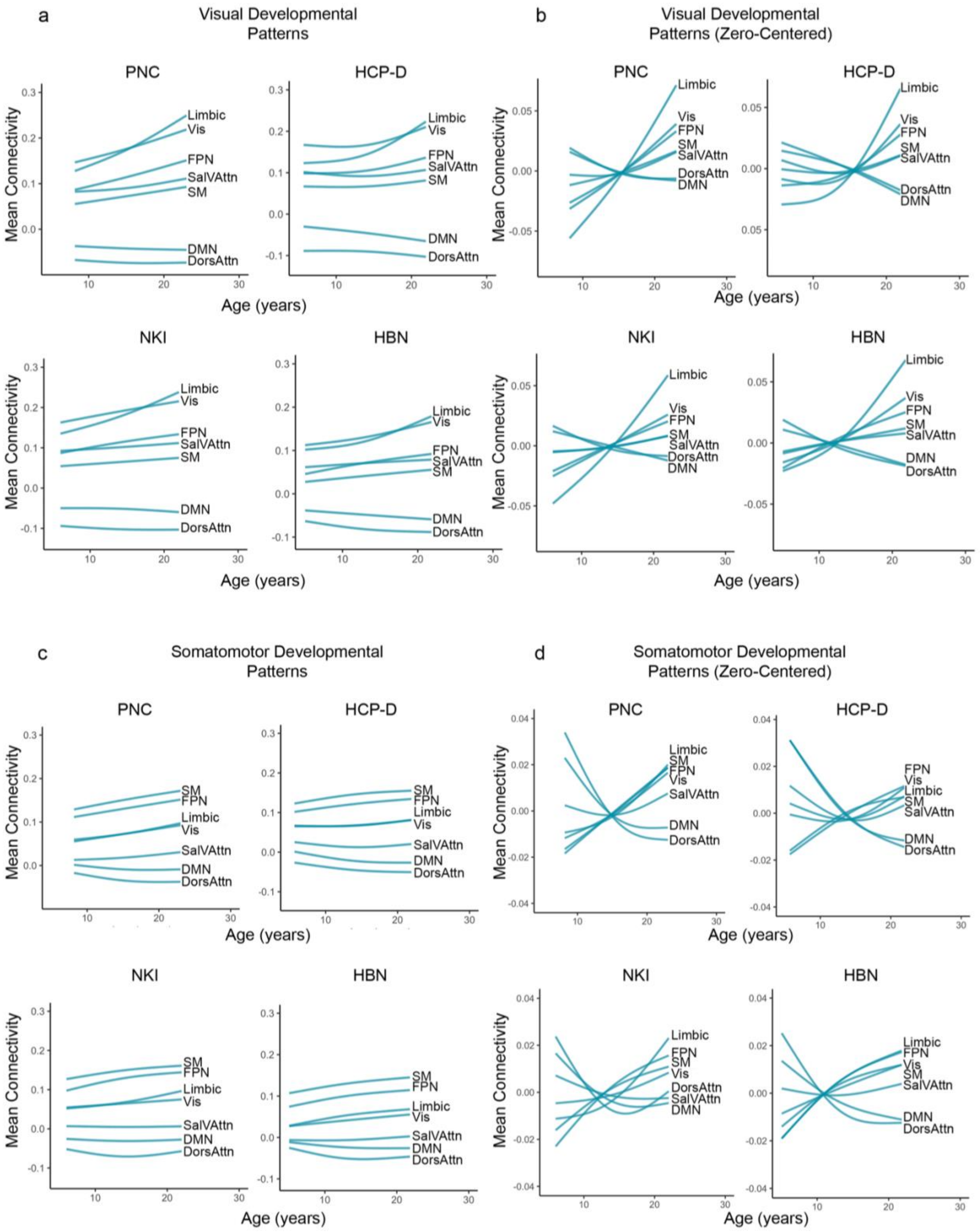

Supplementary Figure 9. Comparing between- and within-network connectivity developmental trajectories in visual and somatomotor networks. For each dataset, the developmental change in mean connectivity of (a-b) visual and (c-d) somatomotor networks to all other networks is plotted. In (a) and (c), predicted mean connectivity generated from generalized additive models fit on each pair of networks

are displayed for each dataset. In (b) and (d), zero-centered developmental trajectories that display directional changes are plotted for each dataset. Visual and somatomotor networks exhibit highly similar developmental changes in connectivity. Networks are partitioned using the Yeo 7 network solution.

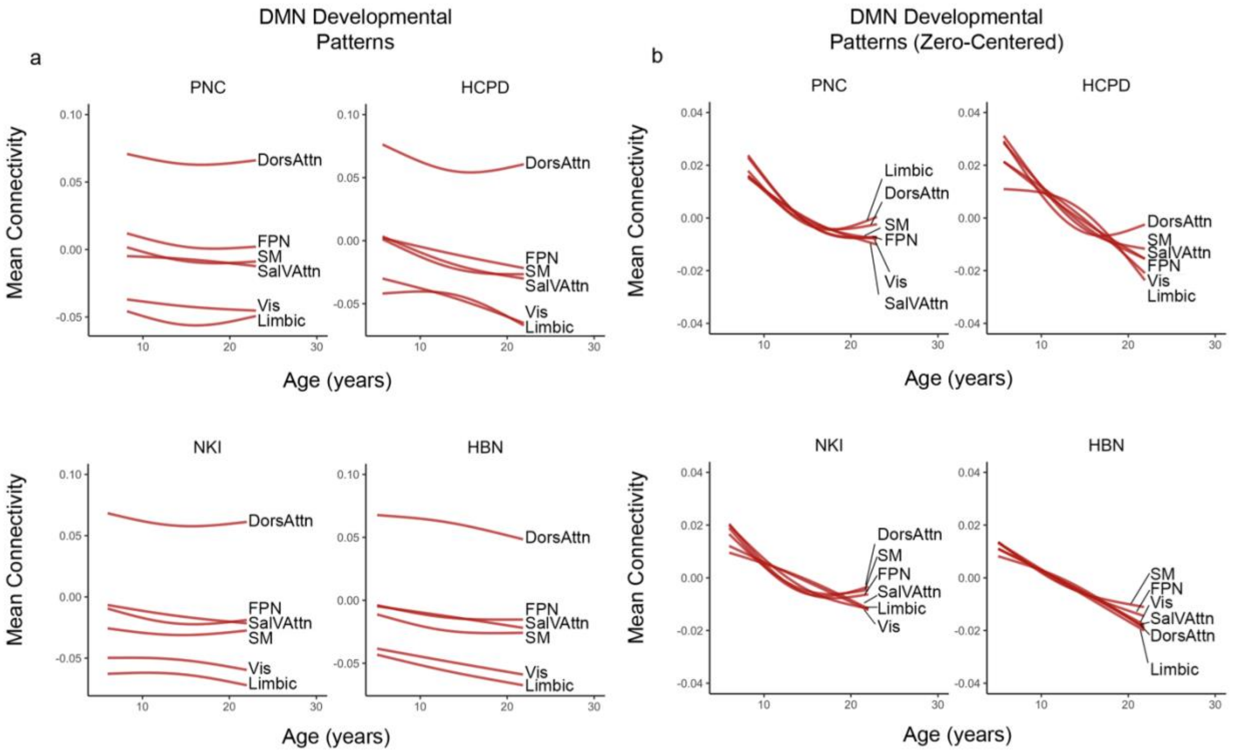

Supplementary Figure 10. Developmental changes in between-network connectivity for default mode network. (a-b) In PNC, NKI, HCP-D, and HBN, default mode network (DMN) decreases in mean connectivity to all other networks with age. Connectivity between DMN and fronto-parietal, salience / ventral attention, somatomotor, visual, and limbic networks tend to be negative or close to zero in childhood and becomes increasingly negative with development. (a) Predicted mean connectivity generated from generalized additive models fit on each pair of networks are displayed for each dataset. (b) Zero-centered developmental trajectories that display directional changes are plotted for each dataset.

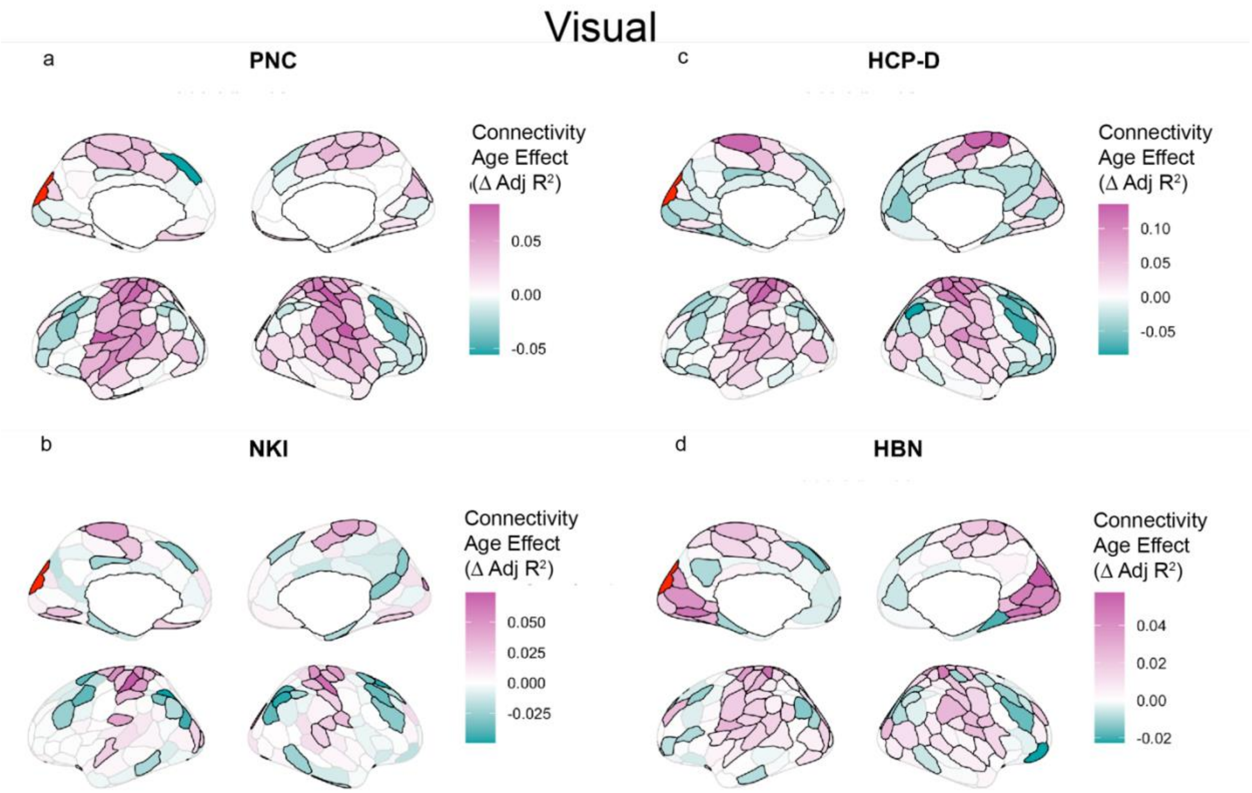

Supplementary Figure 11. Age effect maps of connectivity for a select region from the visual network. Maps are shown for (a) PNC, (b) NKI, (c) HCP-D, and (d) HBN. Age effects of connectivity between the indicated visual seed region (red) and the rest of cortex are shown on the cortical surface with pink indicating increasing connectivity with age and teal indicating decreasing connectivity with age. The visual seed region tends to increase most in connectivity to somatomotor and other visual regions while segregating from DMN regions. All regions outlined in black display significant changes in connectivity ($Q_{FDR} < 0.05$). Results are shown in the Schaefer 200 atlas with a 7-network partition based on the 7 Yeo network solution.

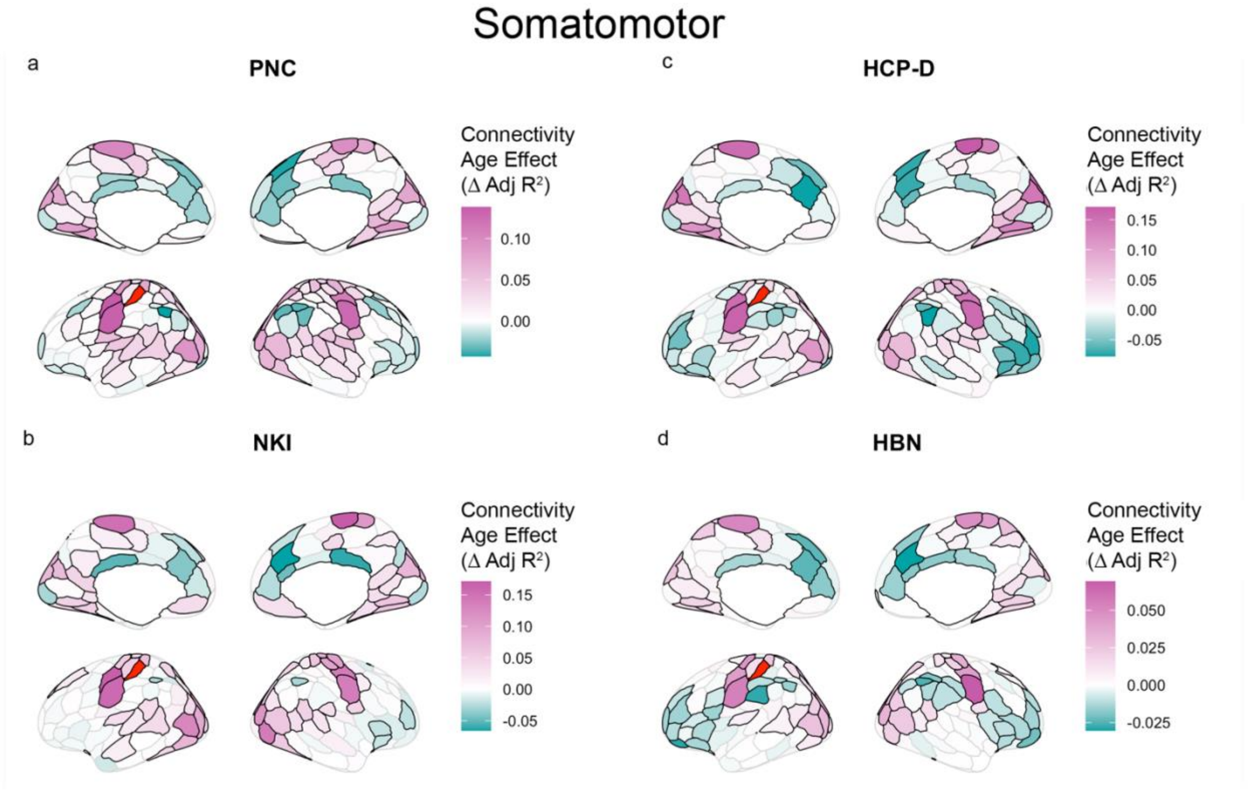

Supplementary Figure 12. Maps of developmental change in connectivity for a seed region from the somatomotor network. Spatial patterns for the somatomotor seed region are generally consistent across (a) PNC, (b) NKI, (c) HCP-D, and (d) HBN. The somatomotor region (red) tends to increase most in connectivity to other somatomotor regions and moderately to visual regions, while segregating from regions within the DMN. Regions outlined in black exhibit significant changes in connectivity ($Q_{FDR} < 0.05$). Results are shown in the Schaefer 200 atlas with a 7-network partition based on the 7 Yeo network solution.

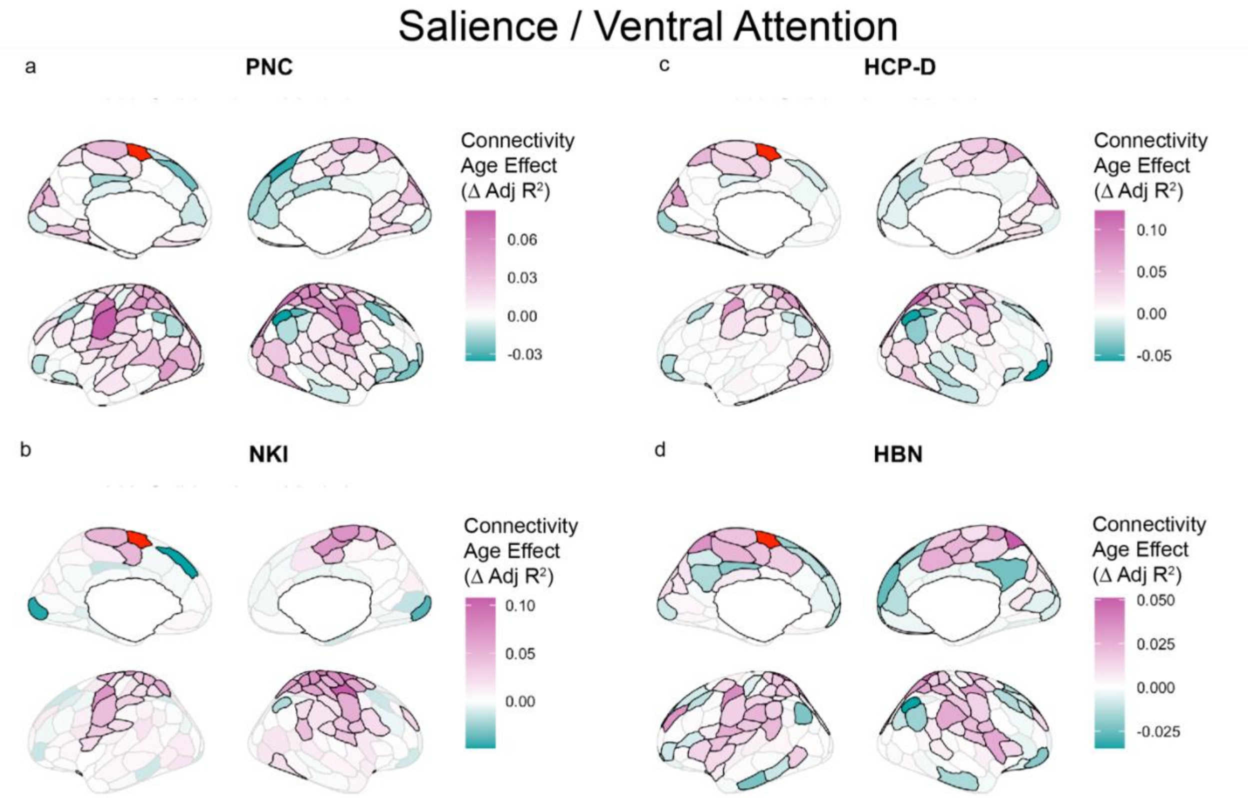

Supplementary Figure 13. Age effect maps for connectivity in a select region from the salience / ventral attention network. Maps are depicted for (a) PNC, (b) NKI, (c) HCP-D, and (d) HBN. The region from the salience / ventral attention network (red) strengthens in connectivity with other salience / ventral attention network regions and with somatomotor regions but decreases in connectivity with DMN. Regions outlined in black exhibit significant changes in connectivity ($Q_{\mathrm{FDR}} < 0.05$). Results are shown in the Schaefer 200 atlas with a 7-network partition based on the 7 Yeo network solution.

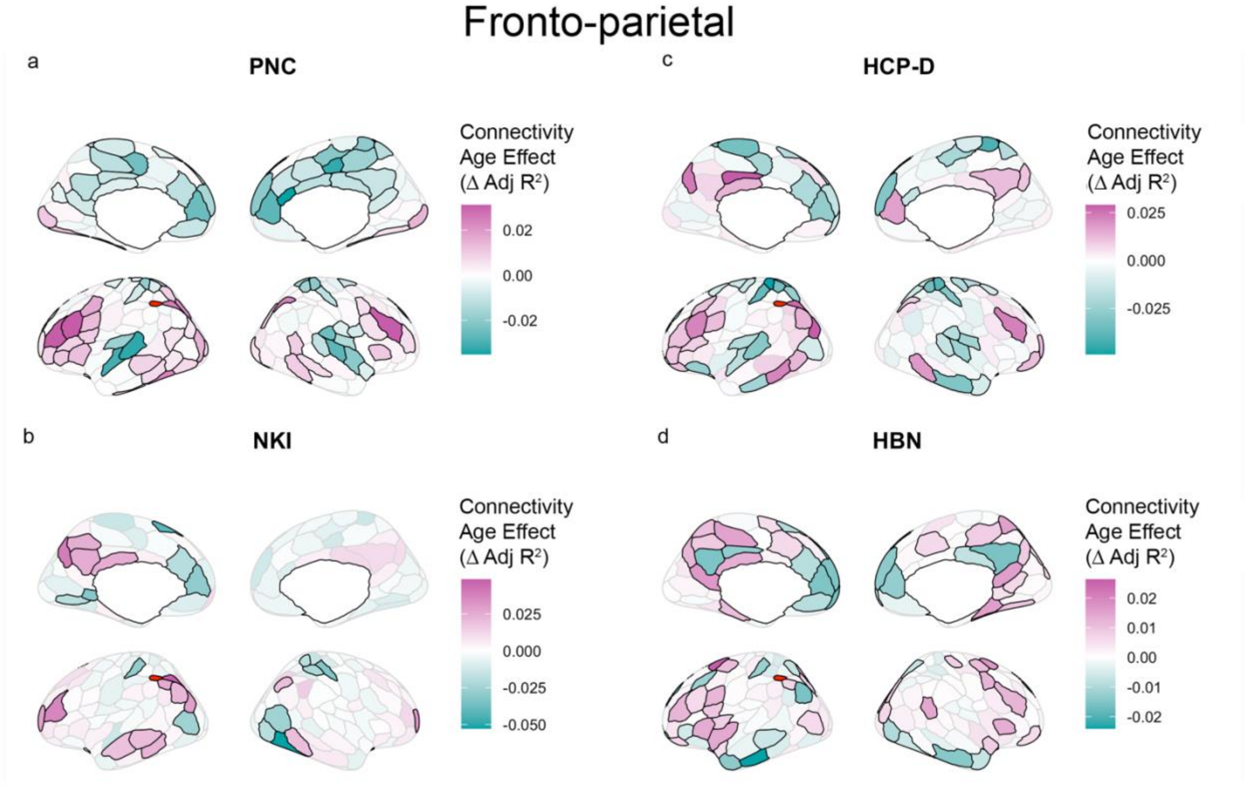

Supplementary Figure 14. Maps of developmental change in connectivity for a seed region from the fronto-parietal network (FPN). Maps are displayed for (a) PNC, (b) NKI, (c) HCP-D, and (d) HBN. This FPN region (red) tends to increase in within-network connectivity and moderately increase in connectivity to diverse regions in the visual, salience / ventral attention, and dorsal attention networks. All regions outlined in black display significant changes in connectivity ($Q_{FDR} < 0.05$). Results are shown in the Schaefer 200 atlas with a 7-network partition based on the 7 Yeo network solution.

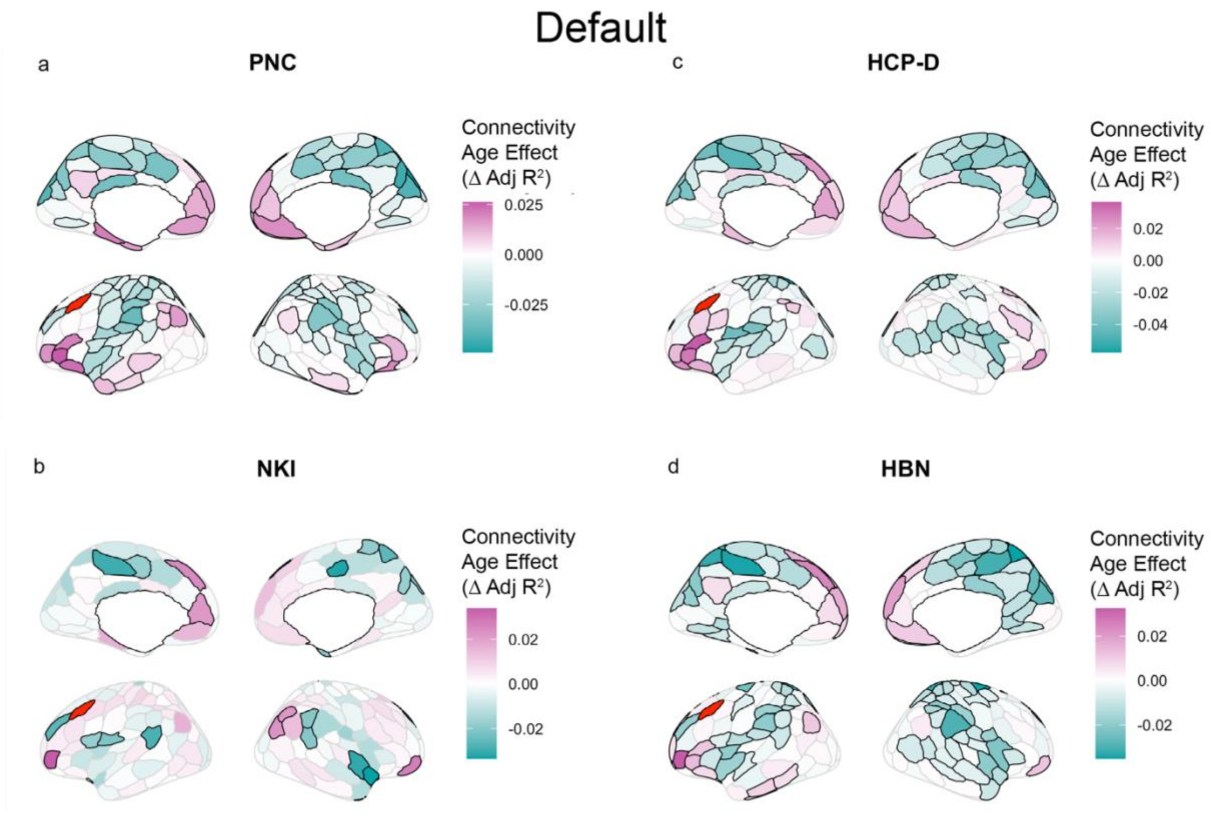

Supplementary Figure 15 Age effect maps of connectivity for a seed region from the default mode network. Maps are displayed for (a) PNC, (b) NKI, (c) HCP-D, and (d) HBN. The default mode network region (red) increases in within-network connectivity while segregating from sensorimotor, salience / ventral attention, dorsal attention, and fronto-parietal regions. Regions outlined in black exhibit significant changes in connectivity ($Q_{FDR} < 0.05$). Results are shown in the Schaefer 200 atlas with a 7-network partition based on the 7 Yeo network solution.

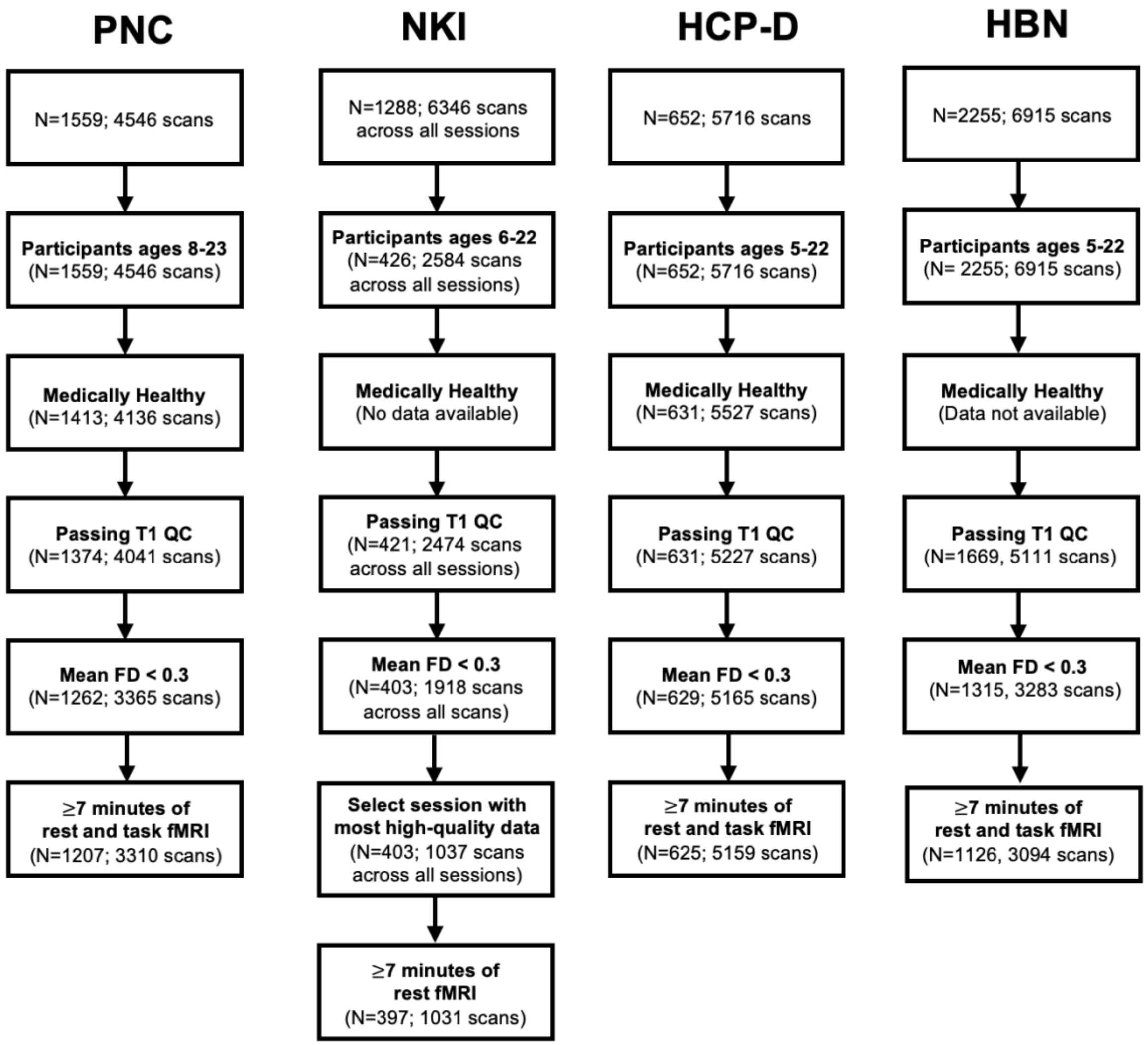

Supplementary Figure 16. Flow diagram depicting sample selection and inclusion and exclusion criteria. Data were taken from four large neuroimaging datasets: Philadelphia Neurodevelopmental Cohort (PNC), Nathan-Kline Institute-Rockland Sample (NKI), Human Connectome Project: Development (HCP-D), and Healthy Brain Network (HBN). We included participants ages 5-23 as available for each dataset. Participants were excluded for medical conditions affecting brain function or gross neurological abnormalities, poor T1 quality, and excessive in-scanner head motion. Furthermore, participants without at least 7 minutes of scan time after concatenating available rest and task fMRI scans were excluded. Note that NKI only collected resting-state fMRI. Furthermore, NKI collected neuroimaging data for each participant over multiple sessions. Scans from the session with the greatest number of surviving scans after head motion exclusion were selected for analysis.

Dataset	# of Scanning Sites	Site	MRI platform
PNC	1	University of Pennsylvania	3T Siemens Trio with 32-channel head coil
NKI	1	Nathan Kline Institute	3T Siemens Trio with 32-channel head coil
HCP-D	4	University of Minnesota, Harvard University, Washington University in St. Louis, and University of California-Los Angeles	3T Siemens Prisma with 32-channel head coil
HBN	4	Staten Island Flagship Research Center	1.5T Siemens Avanto with 32-channel head coil
		Rutgers University Brain Imaging Center	3T Siemens Tim Trio with 32-channel head coil
		CitiGroup Cornell Brain Imaging Center	3T Siemens Tim Trio with 32-channel head coil
		CUNY Advanced Science Research Center	3T Siemens Prisma with a 32-channel head coil

Table S1. Scanning site and MRI platform information for each dataset.

Dataset	T1										Field Map								
	Sequence		TR (ms)	TE (ms)	TI (ms)	Flip angle	FOV (mm^2)	Slice #	Voxel Size (mm)	Acq. Time (mm:ss)	Sequence		TR (ms)	TE (ms)	Flip angle	FOV (mm^2)	Slice #	Voxel Size (mm)	Acq. Time (mm:ss)
PNC	MPRAGE		1810	3.51	1100	9°	180 × 240	160	0.94 × 0.94 ×	3:28	Dual-echo GRE		1000	2.69; 5.27	60°	240 × 240	44	3.8 × 3.8 × 4	-
NKI	MPRAGE		1900	2.52	900	9°	250 × 250	176	1.0 × 1.0 × 1.0	4:18	-		-	-	-	-	-	-	-
HCP-D	MEMPRAGE		2500	1.8. 3.6, 5.4, 7.2 ms	1000	8°	256 × 256	208	0.8 × 0.8 × 0.8	8:22	Spin echo (AP + PA)		8000	66	90°	208 × 208	72	2.0 × 2.0 × 2.0	0:18
HBN	MEMPRAGE	Staten Island	2730	1.6, 3.5, 5.36, 7.22	1000	7°	256 × 256	176	1.0 × 1.0 × 1.0	6:32	Spin echo (AP + PA)	Staten Island	492	3.28 and 8.04	90°	192 × 192	42	3.0 × 3.0 × 3.0	1:05
	MPRAGE	All other sites	2500	3.15	1060	8°	256 × 256	224	0.8 × 0.8 × 0.8	7:19		All other sites	5301	51.2	90°	202 × 202	60	2.4 × 2.4 × 2.4	0:05

MPRAGE: magnetization-prepared rapid acquisition gradient-echo
MEMPRAGE: multi-echo magnetization-prepared rapid acquisition gradient-echo
EPI: echo planar imaging

Table S2. Image acquisition parameters for T1-weighted images and field maps for each dataset.

Dataset	Resting-state fMRI									Task fMRI							
	Sequence		TR (ms)	TE (ms)	Flip angle	FOV (mm²)	Slice #	Voxel Size (mm)	Acq. Time (mm:ss)	Sequence	TR (ms)	TE (ms)	Flip angle	FOV (mm²)	Slice #	Voxel Size (mm)	Acq. Time (mm:ss)
PNC	Singleband EPI		3000	32	90°	192 × 192	46	3.0 × 3.0 × 3.0	6:18	Singleband EPI	3000	32	90°	192 × 192	46	3.0 × 3.0 × 3.0	Emotion ID 10:36
																	N-Back 11:39
NKI	Multiband EPI		645	30	60°	222 × 222	40	3.0 × 3.0 × 3.0	9:46	-	-	-	-	-	-	-	-
			1400		65°	224 × 224	64	2.0 × 2.0 × 2.0	9:35								
	Singleband EPI		2500		80°	216 × 216	38	3.0 × 3.0 × 3.0	5:00								
HCP-D	Multiband EPI		800	37	52°	208 × 208	72	2.0 × 2.0 × 2.0	6:40 (5:15 for participants ages 5-7)	Multiband EPI	800	37	52°	208 × 208	72	2.0 × 2.0 × 2.0	Guessing 3:55 for each run (two runs total)
																	Carit 4:11 for each run (two runs total)
																	Emotion 2:33 (1 run)
HBN	Multi band EPI	Staten Island	1450	40	55	192 × 192	54	2.5 × 2.5 × 2.5	10:18	Multiband EPI	1450	40	55	192 × 192	54	2.5 × 2.5 × 2.5	Movie: Despicable Me 10:08
		All other sites	800	30	31	204 × 204	60	2.4 × 2.4 × 2.4	2 runs, 5:08 each		800	30	31°	204 × 204	60	2.4 × 2.4 × 2.4	Movie: The Present 3:28

Table S3. Image acquisition parameters for resting-state and task fMRI for each dataset.
