# Supplementary material for: Functional connectivity development along the sensorimotor-association axis enhances the cortical hierarchy
Source: Nat Commun. 2024 Apr 25;15:3511. doi: 10.1038/s41467-024-47748-w (PMC11045762; doi:10.1038/s41467-024-47748-w)
Supplement: Supplementary file 3 — Reporting Summary [file 41467_2024_47748_MOESM3_ESM.pdf]

nature portfolio

Corresponding author(s): Theodore D. Satterthwaite, M.D

Last updated by author(s): 2024/02/22

Reporting Summary

Nature Portfolio wishes to improve the reproducibility of the work that we publish. This form provides structure for consistency and transparency in reporting. For further information on Nature Portfolio policies, see our Editorial Policies and the Editorial Policy Checklist.

Statistics

For all statistical analyses, confirm that the following items are present in the figure legend, table legend, main text, or Methods section.

n/a	Confirmed	
☐	☒	The exact sample size (*n*) for each experimental group/condition, given as a discrete number and unit of measurement
☐	☒	A statement on whether measurements were taken from distinct samples or whether the same sample was measured repeatedly
☐	☒	The statistical test(s) used AND whether they are one- or two-sided<br>*Only common tests should be described solely by name; describe more complex techniques in the Methods section.*
☐	☒	A description of all covariates tested
☐	☒	A description of any assumptions or corrections, such as tests of normality and adjustment for multiple comparisons
☐	☒	A full description of the statistical parameters including central tendency (e.g. means) or other basic estimates (e.g. regression coefficient) AND variation (e.g. standard deviation) or associated estimates of uncertainty (e.g. confidence intervals)
☐	☒	For null hypothesis testing, the test statistic (e.g. *F*, *t*, *r*) with confidence intervals, effect sizes, degrees of freedom and *P* value noted<br>*Give P values as exact values whenever suitable.*
☒	☐	For Bayesian analysis, information on the choice of priors and Markov chain Monte Carlo settings
☒	☐	For hierarchical and complex designs, identification of the appropriate level for tests and full reporting of outcomes
☐	☒	Estimates of effect sizes (e.g. Cohen's *d*, Pearson's *r*), indicating how they were calculated

Our web collection on statistics for biologists contains articles on many of the points above.

Software and code

Policy information about availability of computer code

Data collection

Philadelphia Neurodevelopmental Cohort (PNC): scanned on single 3 Tesla Siemens TIM Trio
Nathan Kline Institute-Rockland Sample (NKI): scanned on single 3 Tesla Siemens TIM Trio
Human Connectome Project-Development (HCP-D): scanned on 3 Tesla Siemens Prisma in 4 different sites
Healthy Brain Network (HBN): scanned on 1.5T Siemens Avanto, 3T Siemens Tim Trio, and 3T Siemens Prisma in 4 different sites

Data analysis

Neuroimaging data were processed with containerized software packages available on dockerhub. Functional MRI data were processed with fMRIPrep (https://hub.docker.com/r/nipreps/fmriprep/tags) and XCP-D (https://hub.docker.com/r/pennlinc/xcp_abcd/tags).

The following versions of fMRIPrep were used for each dataset: 20.2.3 (PNC and NKI) and 22.0.2 (HCP-D and HBN). The following versions of XCP-D were used for each dataset: 0.0.8 (NKI), 0.3.0 (HCP-D), and 0.3.2 (PNC and HBN).

Internal operations of fMRIPrep 20.2.3 use the following software: Advanced Normalization Tools 2.3.3, Nipype 1.6.1, FSL 5.0.9, FreeSurfer 6.0.1, and AFNI 20160207.

Internal operations of fMRIPrep 22.0.2 use the following software: Advanced Normalization Tools 2.3.3, Nipype 1.6.1, FSL 6.0.5.1, and FreeSurfer 7.2.0, and AFNI 20160207.

Internal operations of XCP-D 0.0.8 use Scikit-Learn 0.24.2; XCP-D 0.3.0 uses Scikit-Learn 1.1.3; XCP-D 0.3.2 used nilearn 0.9.2.

Following image processing, all subsequent analyses and statistics were conducted in R 4.1.2 (https://www.r-project.org) using original

analysis code and Connectome Workbench 1.5.0 tools (https://www.humanconnectome.org/software/get-connectome-workbench).

Generalized additive models (GAM) were fit using the mgcv package (version 1.8.39) in R. Fitted values from the GAMs were computed using the gratia package (version 0.7.0) in R. Harmonization was completed using the CombatFamily package (version 0.1.0) in R. Statistical significance using spin-based spatial permutation tests used the 'rotate_parcellation' algorithm in R (https://github.com/frantisekvasa/rotate_parcellation).

A detailed description of the original code can be found at https://github.com/PennLINC/network_replication and a guide to code implementation can be found at https://pennlinc.github.io/network_replication.

For manuscripts utilizing custom algorithms or software that are central to the research but not yet described in published literature, software must be made available to editors and reviewers. We strongly encourage code deposition in a community repository (e.g. GitHub). See the Nature Portfolio guidelines for submitting code & software for further information.

Data

Policy information about availability of data

All manuscripts must include a data availability statement. This statement should provide the following information, where applicable:

- Accession codes, unique identifiers, or web links for publicly available datasets
- A description of any restrictions on data availability
- For clinical datasets or third party data, please ensure that the statement adheres to our policy

This paper analyzes publicly available data from four datasets: the PNC, accessible from the Database of Genotypes and Phenotypes (phs000607.v3.p2) at https://www.ncbi.nlm.nih.gov/projects/gap/cgi-bin/study.cgi?study_id=phs000607.v3.p2.

NKI is available at https://openneuro.org/datasets/ds001021/versions/1.0.0.

HCP-D is available for download through the NIMH Data Archive (https://nda.nih.gov/).

HBN is accessible through http://fcon_1000.projects.nitrc.org/indi/cmi_healthy_brain_network/.

Furthermore, analyses utilized publicly available cortical atlases: the HCP multimodal atlas (downloaded from https://github.com/PennLINC/xcp_d/blob/665840c5ec5586afe31513c57c1f563eaeaba715/xcp_d/data/ciftiatlas/glasser_space-fsLR_den-32k_desc-atlas.dlabel.nii), Schaefer 200 atlas (https://github.com/PennLINC/xcp_d/blob/665840c5ec5586afe31513c57c1f563eaeaba715/xcp_d/data/ciftiatlas/schaefer_space-fsLR_den-32k_desc-200Parcels17Networks_atlas.dlabel.nii), Schaefer 400 atlas (https://github.com/PennLINC/xcp_d/blob/665840c5ec5586afe31513c57c1f563eaeaba715/xcp_d/data/ciftiatlas/schaefer_space-fsLR_den-32k_desc-400Parcels17Networks_atlas.dlabel.nii), and Gordon atlas (https://github.com/PennLINC/xcp_d/blob/665840c5ec5586afe31513c57c1f563eaeaba715/xcp_d/data/ciftiatlas/gordon_space-fsLR_den-32k_desc-atlas.dlabel.nii).

The sensorimotor-association axis was downloaded from https://github.com/PennLINC/S-A_ArchetypalAxis.

Source Data generated in this study have been deposited in the Zenodo database under accession https://doi.org/10.5281/zenodo.10818786.

Research involving human participants, their data, or biological material

Policy information about studies with human participants or human data. See also policy information about sex, gender (identity/presentation), and sexual orientation and race, ethnicity and racism.

Reporting on sex and gender	All datasets used in this study reported on self-reported participant sex as a biological attribute (intersex was not assessed). Sex was considered in all statistical analyses: we conducted analyses that included sex as a covariate. PNC included 1207 individuals with 646 females and 561 males. NKI included 397 individuals, with 186 females and 211 males. HCP-D included 625 participants, 337 of which were female and 288 were male. HBN had a sample of 1126, with 439 females and 687 males.
Reporting on race, ethnicity, or other socially relevant groupings	For PNC, the study sample demographics include an age range of 8 to 23 years (mean age = 15.4 ± 3.5 years) and a race and ethnicity distribution (self-reported) that was 0.9% Asian, 42.5% Black or African American, 10.9% identifying as multiracial or with individuals identifying as American Indian or Alaska Native, Hispanic or Latino, or Native Hawaiian or Other Pacific Islander, and 45.7% White. There were no participants missing racial data.<br><br>For NKI, the study sample demographics include an age range of 6 to 22 years (mean age = 14.5 ± 4.4 years) and a race and ethnicity distribution (self-reported) that was 8.5% Asian, 20.7% Black or African American, 2.8% identifying as multiracial or with individuals identifying as American Indian or Alaska Native, Hispanic or Latino, or Native Hawaiian or Other Pacific Islander, and 65.0% White. Twelve participants (3%) were missing racial data.<br><br>For HCP-D, the study sample demographics include an age range of 5 to 22 years (mean age = 14.5 ± 4.1 years) and a race and ethnicity distribution (self-reported) that was 7.7% Asian, 11% Black or African American, 15.5% identifying as multiracial or with individuals identifying as American Indian or Alaska Native, Hispanic or Latino, or Native Hawaiian or Other Pacific Islander, and 63.2% White. Sixteen participants (2.6%) were missing racial data.<br><br>For HBN, the study sample demographics include an age range of 5 to 22 years (mean age = 11.6 ± 3.5 years) and a race and ethnicity distribution (self-reported) that was 2.9% Asian, 12.3% Black or African American, 27.3% identifying as multiracial or with individuals identifying as American Indian or Alaska Native, Hispanic or Latino, or Native Hawaiian or Other Pacific

	Islander, and 44.2% White. One hundred and forty-nine participants (13.2%) were missing racial data.
Population characteristics	See the above two boxes for age, sex, and race characteristics.
Recruitment	PNC: participants were recruited for this study had previously been assessed at the Center for Applied Genomics at the Children's Hospital of Philadelphia and the University of Pennsylvania and had provided written consent to be re-contacted for future research. All participants were from the greater Philadelphia area and were scanned at a single site.<br><br>NKI: Program staff targeted multiple educational and recruitment outreach opportunities in Rockland County, New York to collaborate with the community for recruitment. Healthy participants were recruited through study recruitment booths at community events including local street fairs and the NKI Neuroscience Education Day. All participants were scanned at a single site.<br><br>HCP-D: healthy participants were recruited and scanned across four sites: University of Minnesota, Harvard University, Washington University in St. Louis, and University of California-Los Angeles.<br><br>HBN: HBN utilized a community-referred recruitment model. This study used advertisements to encourage participation of families who have concerns about psychiatric symptoms in their child. Participants were recruited and scanned at four different sites: Staten Island Flagship Research Center, Rutgers University Brain Imaging Center, CitiGroup Cornell Brain Imaging Center, and CUNY Advanced Science Research Center.
Ethics oversight	PNC: All study procedures were approved by the Institutional Review Boards of both the University of Pennsylvania and the Children's Hospital of Philadelphia.<br><br>NKI: All study procedures were approved by the Institutional Review Board at the Nathan Kline Institute.<br><br>HCP-D: All study procedures were approved by a central Institutional Review Board at Washington University in St. Louis.<br><br>HBN: All study procedures were approved by the Chesapeake Institutional Review Board.

Note that full information on the approval of the study protocol must also be provided in the manuscript.

Field-specific reporting

Please select the one below that is the best fit for your research. If you are not sure, read the appropriate sections before making your selection.

☒ Life sciences ☐ Behavioural & social sciences ☐ Ecological, evolutionary & environmental sciences

For a reference copy of the document with all sections, see nature.com/documents/nr-reporting-summary-flat.pdf

Life sciences study design

All studies must disclose on these points even when the disclosure is negative.

Sample size	For all datasets, all of the neuroimaging data made available was considered for inclusion in analysis. Sample size was not chosen based on a pre-specified power analysis.
Data exclusions	Age Exclusion<br>Participants ages 5-23 were included in our study as available per dataset. In the PNC, HCP-D, and HBN, no additional participants were excluded since all participants were within the age window studied. Data from n = 844 individuals were excluded from NKI's original lifespan sample of n = 1,268 due to participants being outside the desired age window.<br><br>Medical Exclusion<br>Exclusion criteria included the presence of medical conditions affecting brain function (when assessed) or gross neurological abnormalities, as well as MRI scanner contraindications. In the PNC, n = 146 were excluded from the sample of n = 1,559, and n = 21 participants were excluded from the original sample of n = 652 in HCP-D. Medical exclusion data was not available for NKI and HBN.<br><br>T1 Exclusion<br>We excluded low-quality T1-weighted images that did not survive manual quality assurance (when possible, based on available data). For the PNC, three highly trained raters provided manual ratings of whether images were usable or not based on artifacts. Thirty-nine participants were excluded for T1 quality in the PNC. For NKI and HBN, the Swipes for Science web application was used to perform visual quality control. Raters chose to pass or fail an image based on visual inspection of the general quality of the image and the blurriness between the white and gray matter boundary. An additional n = 5 participants were excluded from NKI due to poor T1 quality. For HBN, 586 participants were excluded for T1 quality. No additional participants were excluded in HCP-D; T1 exclusion was completed by the team that collected the data.<br><br>fMRI Motion Exclusion<br>We excluded task and rest fMRI scans with high in-scanner head motion, as defined as mean framewise displacement ≥ 0.3. Participants were excluded at this stage if all fMRI scans for a given participant failed head motion exclusion. For the PNC, an additional n = 112 participants were excluded for high in-scanner motion. In NKI, n = 18 participants were excluded; in HCP-D, n = 2 participants were excluded; and in HBN, n = 354 participants were excluded for high in-scanner head motion. In PNC, HCP-D, and HBN, task and rest scans that survived head motion exclusion were concatenated to maximize scan time. NKI collected only resting-state fMRI and was not concatenated. Note that because NKI had multiple sessions of MRI scans available, we utilized scans from the session with the greatest number of scans surviving T1 and head

	motion exclusion for subsequent analyses.
Replication	An in-depth code review and replication of the first author's study code was performed once by the study's second author. Every analysis was successfully and independently reproduced internally. All code needed to reproduce experimental findings is detailed in the manuscript's code availability statement. A guide to implementing this code is additionally provided at https://pennlinc.github.io/ network_replication
Randomization	No experimental groups or conditions were included in this study; thus no randomization was performed.
Blinding	No group allocation was conducted, thus blinding was not needed.

Reporting for specific materials, systems and methods

We require information from authors about some types of materials, experimental systems and methods used in many studies. Here, indicate whether each material, system or method listed is relevant to your study. If you are not sure if a list item applies to your research, read the appropriate section before selecting a response.

Materials & experimental systems

n/a	Involved in the study
☒	☐ Antibodies
☒	☐ Eukaryotic cell lines
☒	☐ Palaeontology and archaeology
☒	☐ Animals and other organisms
☒	☐ Clinical data
☒	☐ Dual use research of concern
☒	☐ Plants

Methods

n/a	Involved in the study
☒	☐ ChIP-seq
☒	☐ Flow cytometry
☐	☒ MRI-based neuroimaging

Magnetic resonance imaging

Experimental design

Design type

Eyes-open resting state fMRI was collected for all studies. Lengths of rest scans can be found under Sequence & imaging parameters.

Design specifications

Eyes-open resting state fMRI was collected for all studies. Lengths of rest scans can be found under Sequence & imaging parameters.

The following outlines the task fMRI design specifications.

PNC: The two tasks performed were the n-back task and the emotion identification task. The n-back task involved presentation of complex geometric figures for 500ms, followed by a fixed interstimulus interval of 2500ms. This occurred under three conditions: 0-back, 1-back, and 2-back, producing different levels of working memory load. Each condition consisted of a 20-trial block (60 s); each condition was repeated over three blocks. Total task duration was 11 min 36 s (24 s rest while fixation crosshair was shown between each of the three blocks for each condition, with 2500 ms as ISI).

In the emotion identification task, participants viewed 60 faces displaying neural, happy, sad, angry or fearful expressions and were asked to label the emotion. Each emotional face was displayed for 5.5 seconds followed by a variable interstimulus interval of 0.5 to 18.5 seconds. Total task duration was 10 min 30 s.

NKI: Only resting-state data was collected.

HCP-D:
Reward Magnitude ("guessing") task aims to measure neural responses to gains and losses of different magnitudes. For each trial, participants view a guess cue indicating upcoming high or low stake trials, a jittered interstimulus interval, and then view feedback indicating whether they are rewarded or lose money. Number of runs = 2 runs; trials per run = Each run has 6 blocks of 4 trials each for a total of 24 trials per run. Trials consistent of reward and loss trials for high and low stake conditions; duration = 7 min 27 s; interval between trials = 2 s jitter ITI.

In the Inhibitory control (or Conditioned Approach Response Inhibition Task, CARIT), participants view shape stimuli. Participants are instructed to press a button as quickly as possible to every shape except for the circle and the square ('no-go' shapes). Number of runs = 2 runs; trials per run = 24 NoGo's per run and 68 Go's per run (for total of 92 trials per run); duration = 8 sec countdown + 3min 51.6 sec per task run, total: 239.6s + .4s end fix per run (4:00min), for 8 minutes total; interval between trials = 1.0 to 4.5s jitter ITI.

During the Emotion task, participants see images of either emotional faces or shapes (3 total), one at the top and two at the bottom of the screen. The face stimuli depict angry or fearful expressions. Participants are told to press the left button if the left-hand image on the bottom of the screen matches the top image, and to press the right button if the

right-hand image on the bottom of the screen matches the top image. Number of runs = 1 run; trials per session = 6 blocks per run, and each block has 6 trials. Each trial presents 3 stimuli; duration = 8 sec countdown + 2 min 22 s per task run, total: 2 min 36 s; interval between trials = 1.0 s ITI.

HBN: Participants viewed 3 minutes of movie 1 (The Present) and 10 minutes of movie 2 (Despicable Me).

Behavioral performance measures

Performance measures were not used.

Acquisition

Imaging type(s)

T1-weighted MRI, resting-state and task functional MRI, B0 field map

Field strength

3 Tesla for all scanners across datasets except for Staten Island Flagship Research Center (HBN), which had a field strength of 1.5 Tesla.

Sequence & imaging parameters

All information can be found in Supplementary Tables S1-3.
PNC: T1-weighted MRI: T1-weighted structural images were acquired with a magnetization-prepared rapid acquisition gradient-echo (MPRAGE) sequence with the following parameters: repetition time = 1810 ms, echo time = 3.51 ms, inversion time = 1100 ms, flip angle = 9 degrees, field of view = 180 x 240 mm, matrix = 192 x 256, slice number = 160, voxel resolution = 0.94 x 0.94 x 1 mm, acquisition time = 3 min 28 s. Resting-state functional MRI: Resting-state functional images were acquired with a singleband EPI sequence with the following parameters: repetition time = 3 s, echo time = 32 ms, flip angle = 90 degrees, field of view = 192 x 192 mm, matrix = 64 x 64, slice number = 46, voxel resolution = 3 mm3, acquisition time = 6 min 18 s. Task functional MRI: Task functional images were acquired with a singleband EPI sequence with the following parameters: repetition time = 3000 ms, echo time = 32 ms, flip angle = 90 degrees, field of view = 192 x 192 mm, slice number = 46, voxel resolution = 3 mm3, acquisition time = 10 min 36 s (Emotion ID) and 11 min 39 s (N-back). B0 field map: B0 field maps were acquired to enable susceptibility distortion correction of functional images. Field maps were acquired using a dual-echo, gradient-recalled echo (GRE) sequence with the following parameters: repetition time = 1000 ms, echo time 1 = 2.69 ms, echo time 2 = 5.27 ms, flip angle = 60 degrees, field of view = 240 x 240 mm, matrix = 64 x 64, slice number = 44, voxel resolution = 3.8 x 3.8 x 4 mm.

NKI: T1-weighted MRI: T1-weighted structural images were acquired with a magnetization-prepared rapid acquisition gradient-echo (MPRAGE) sequence with the following parameters: repetition time = 1900 ms, echo time = 2.52 ms, inversion time = 900 ms, flip angle = 9 degrees, field of view = 250 x 250 mm, slice number = 176, voxel resolution = 1 mm3, acquisition time = 4 min 18 s.Resting-state functional MRI: Resting-state functional images were acquired with multiband EPI sequence with the following parameters: repetition time = 645 ms and 1400 ms; echo time = 30 ms (for both TR's), flip angle = 60 and 65 degrees respectively, field of view = 222 x 222 mm and 224 x 224 mm, slice number = 40 and 64, voxel resolution = 3 mm3 and 2 mm3, respectively, acquisition time = 9 min 46s and 9 min 35 s respectively. Singleband EPI sequence was additionally used to collect data with the following parameters: repetition time = 2500 ms; echo time = 30 ms, flip angle = 80 degrees, field of view = 216 x 216 mm, slice number = 38, voxel resolution = 3 mm3, acquisition time = 5 min. Task functional MRI: No task fMRI was collected for NKI. B0 field map: No field maps were acquired for NKI.

HCP-D: T1-weighted MRI: T1-weighted structural images were acquired with a multi-echo MPRAGE sequence with the following parameters: repetition time = 2500 ms, echo time 1 = 1.8 ms, echo time 2 = 3.6 ms, echo time 3 = 5.4 ms, echo time 4 = 7.2 ms, inversion time = 1000 ms, flip angle = 8 degrees, field of view = 256 x 256 mm, slice number = 208, voxel resolution = 0.8 mm3, acquisition time = 6 min 54 s. Resting-state functional MRI: Resting-state functional images were acquired with a multiband EPI sequence with the following parameters: repetition time = 800 ms, echo time = 37 ms, flip angle = 52 degrees, field of view = 208 x 208 mm, slice number = 72, voxel resolution = 2 mm3, acquisition time = 6 min 40 s (5 min 15 s for participants ages 5-7). Task functional MRI: Task functional images were acquired with a multiband EPI sequence with the following parameters: repetition time = 800 ms, echo time = 37 ms, flip angle = 52 degrees, field of view = 208 x 208 mm, slice number = 72, voxel resolution = 2 mm3, acquisition time = 3 min 55 s (Guessing), 4 min 11 s (Carit), 2 min 33 s (Emotion). B0 field map: B0 field maps were acquired to enable susceptibility distortion correction of functional images. Field maps were acquired using spin echo sequence with the following parameters: repetition time = 8000 ms, echo time = 66 ms, flip angle = 90 degrees, field of view = 208 x 208 mm, slice number = 72, voxel resolution = 2 x 2 x 2 mm, acquisition time = 18 s.

HBN: T1-weighted MRI: T1-weighted structural images were acquired with a MEMPRAGE sequence with the following parameters at the Staten Island Flagship Research Center: repetition time = 2730 ms, echo time 1= 1.6 ms, echo time 2= 3.5 ms, echo time 3= 5.36 ms, echo time 4= 7.22 ms, inversion time = 1000 ms, flip angle = 7 degrees, field of view = 256 x 256 mm, slice number = 176, voxel resolution = 1mm3, acquisition time: 6 min 32 s. T1-weighted structural images were acquired with MPRAGE sequence with the following parameters at all other sites: repetition time = 2500 ms, echo time = 3.15 ms, inversion time = 1060 ms, flip angle = 8 degrees, field of view = 256 x 256 mm, slice number = 224, voxel resolution =0.8 mm3, acquisition time = 7 min 19 s. Resting-state functional MRI: Resting-state functional images were acquired with a multi-slice echo planar imaging sequence with the following parameters at the Staten Island Flagship Research Center: repetition time = 1450 ms, echo time = 40 ms, flip angle = 55 degrees, field of view = 192 x 192 mm, slice number = 54, voxel resolution = 2.5 mm3, acquisition time: 10 min 18 s. Resting-state functional images were acquired at all other sites with the following parameters: repetition time = 800 ms, echo time = 30 ms, flip angle = 31 degrees, field of view = 204 x 204 mm, slice number = 60, voxel resolution = 2.4 mm3, acquisition time = 2 runs of 5 min 8 s each. Task functional MRI: Task functional images were acquired with a multiband echo planar imaging sequence with the following parameters at the Staten Island Flagship Research Center: repetition time = 1450 ms, echo time = 40 ms, flip angle = 55 degrees, field of view = 192 x 192 mm, slice number = 54, voxel resolution = 2.5 mm3, acquisition time = 10 min 8s (Despicable Me) and 3 min 28 s (The Present). Task functional images acquired with a multiband echo planar imaging sequence at all other sites with the following parameters: repetition time = 800 ms, echo time = 30 ms, flip angle = 31 degrees, field of view = 204 x 204 mm, slice number = 60, voxel resolution = 2.4 mm3,

acquisition time = same as for Staten Island Flagship Research Center. B0 field map: B0 field maps were acquired to enable susceptibility distortion correction of functional images. Field maps were acquired using spin echo sequence with the following parameters at the Staten Island Flagship Research Center: repetition time = 492 ms, echo time 1= 3.28 ms, echo time 2 = 8.04 ms, flip angle = 90 degrees, field of view = 192 x 192 mm, slice number = 42, voxel resolution = 3 mm3, acquisition time = 1 min 5 s. Field maps at all other sites were acquired with the following parameters: repetition time = 5301 ms, echo time = 51.2 ms, flip angle = 90 degrees, field of view = 202 x 202 mm, slice number = 60, voxel resolution = 2.4 mm3, acquisition time = 5 s.

Area of acquisition

A whole-brain scan was acquired

Diffusion MRI ☐ Used ☒ Not used

Preprocessing

Preprocessing software

Preprocessing of T1-weighted images and functional MRI timeseries used fMRIPrep 20.2.3 (PNC and NKI) and 22.0.2 (HCP-D and HBN). A newer release of fMRIPrep was used for HCP-D and HBN to allow for topup-based susceptibility distortion correction given the acquisition of reverse phase encoding directions. Following pre-processing with fMRIPrep, post-processing utilized XCP-D.

Normalization

Structural images underwent correction for intensity non-uniformity with N4BiasFieldCorrection from ANTs 2.3.3, skull-stripping with a Nipype implementation of ANTs brain extraction workflow, and brain tissue segmentation with fast FSL 5.0.9 (PNC and NKI) and 6.0.5.1 (HCP-D and HBN). Brain surfaces were then reconstructed using FreeSurfer 6.0.1 (PNC and NKI) and 7.2.0 (HCP-D and HBN). Volume-based spatial normalization of the T1-weighted image to two standard spaces (MNI152NLin6Asym, MNI152NLin2009cAsym) was performed through nonlinear registration with ANTs.

Normalization template

Data were normalized to the MNI152 T1 template during preprocessing. BOLD timeseries were ultimately projected onto the fsLR cortical surface (32k vertices per hemisphere) for nuisance regression.

Noise and artifact removal

BOLD timeseries were first corrected for head motion (six rotation and translation parameters estimated with FSL mcflirt) and susceptibility distortions (using the B0 field map) in fMRIPrep. fMRIPrep was also used to estimate the following 36 confounds from the preprocessed timeseries: six head motion parameters; three region-wise global signals (mean cerebrospinal fluid, white matter, and whole brain signals); temporal derivatives of the six head motion parameters and the three global signal estimates; and quadratic terms for the motion parameters, tissue signals, and their temporal derivatives.

These confound matrices were utilized within xcp_d 0.0.8, 0.3.0 and 0.3.2 which is an extension of the top-performing eXtensible Connectivity Pipeline (XCP) Engine specifically developed to mitigate motion-related artifacts and noise in resting-state functional MRI data from developmental samples. With xcp_d, preprocessed functional timeseries on the fsLR cortical surface underwent nuisance regression using the 36 confounds listed above. Confounds were regressed using linear regression as implemented in Scikit-Learn 0.24.2 (NKI), Scikit-Learn 1.1.3 (HCP-D), or nilearn 0.9.2 (PNC and HBN).

Volume censoring

BOLD data were despiked with 3dDespike command from XCP-D.

Statistical modeling & inference

Model type and settings

To model both linear and non-linear associations between functional connectivity metrics and age, generalized additive models (GAM) were fit using the mgcv package in R. GAMs were fit for each parcellated cortical region with a given functional connectivity metric (e.g., functional connectivity strength) as the dependent variable, age as a smooth term, and both sex and in-scanner motion as linear covariates. In-scanner head motion was quantified as the mean framewise displacement averaged across all functional runs included for each subject. That is, the average mean framewise displacement of the concatenated task and rest scans surviving T1 and head motion exclusion was used as a covariate. Age was modeled using thin plate regression splines as the smooth term basis set with the maximum basis complexity (k) set to 3 to avoid overfitting. This basis complexity consistently resulted in the lowest model Akaike information criterion (AIC) across cortical regions and datasets. The GAM smooth term for age produces a smooth function (or spline) resulting from a linear combination of weighted basis functions. This spline represents a given region's developmental trajectory for a functional connectivity metric. To examine the spatial distribution of FC strength at specific ages, we generated fitted values of FC strength from the GAM at ages 8, 14, and 22 using the 'fitted_values' function in the gratia package.

Effect(s) tested

To quantify the age effect as in prior work, for each brain region or edge, the effect size of age-related change was quantified by the change in adjusted R2 (ΔR2adj) between a full model and reduced model with no age term. The significance of the association between the functional connectivity metric and age was assessed using analysis of variance (ANOVA) to compare the full and reduced models. To characterize the direction of the effect (increasing or decreasing functional connectivity with age), we evaluated the sign of the age coefficient from an equivalent linear model. Multiple comparisons were controlled for with false discovery rate (FDR) correction; Q<0.05.

Specify type of analysis: ☒ Whole brain ☐ ROI-based ☐ Both

Statistic type for inference

(See Eklund et al. 2016)

Analyses were conducted on a region-wise basis across the cortical surface.

Correction

False discovery rate (FDR) correction was applied when statistical tests were conducted across all cortical regions.

Models & analysis

n/a	Involved in the study
☐	☒ Functional and/or effective connectivity
☒	☐ Graph analysis
☒	☐ Multivariate modeling or predictive analysis

Functional and/or effective connectivity

Functional connectivity strength was computed as the mean edge strength between a given region and all other cortical regions. Individual edge strength was characterized by the Pearson correlation between timeseries for each pair of regions.
